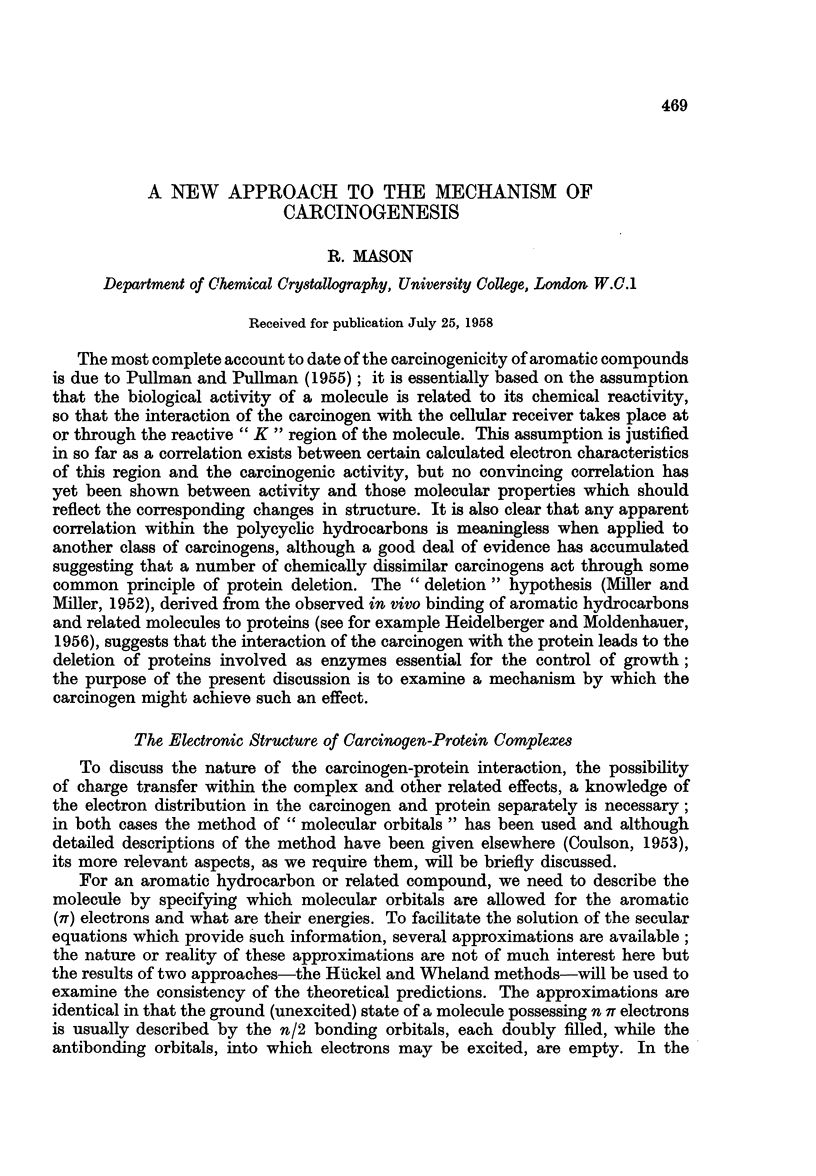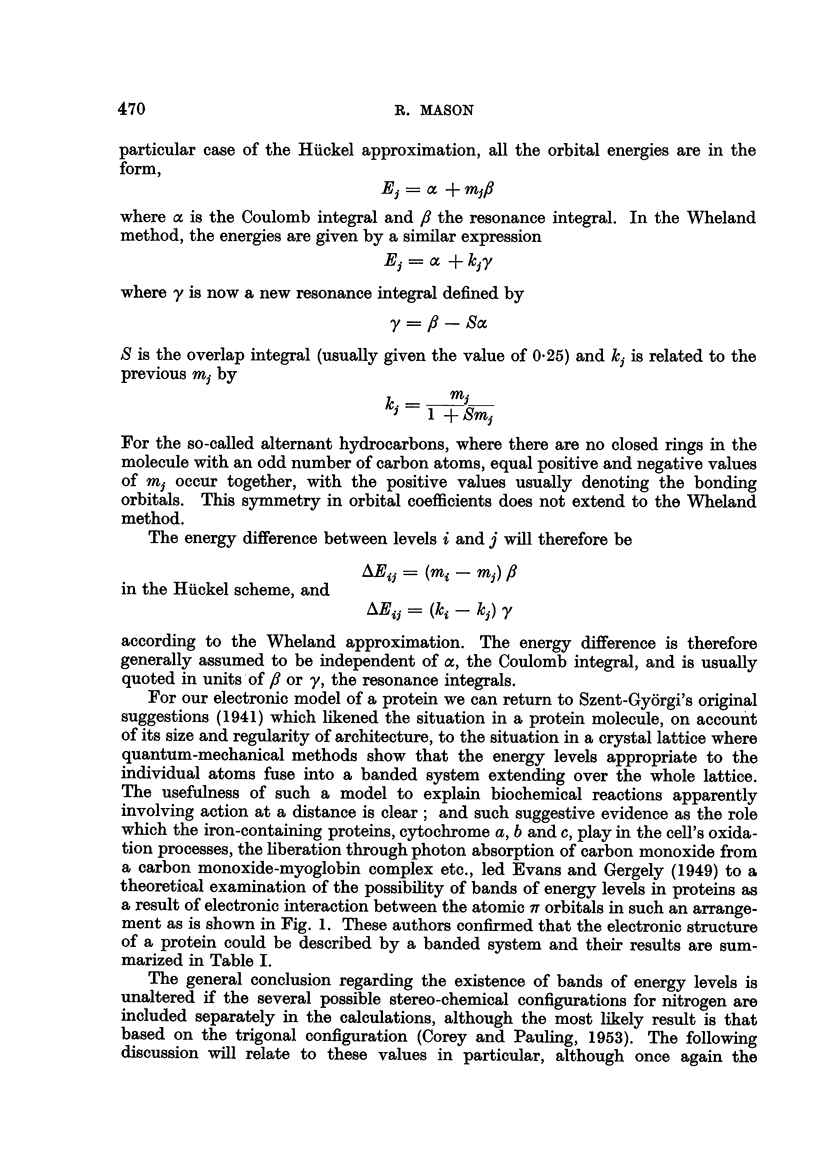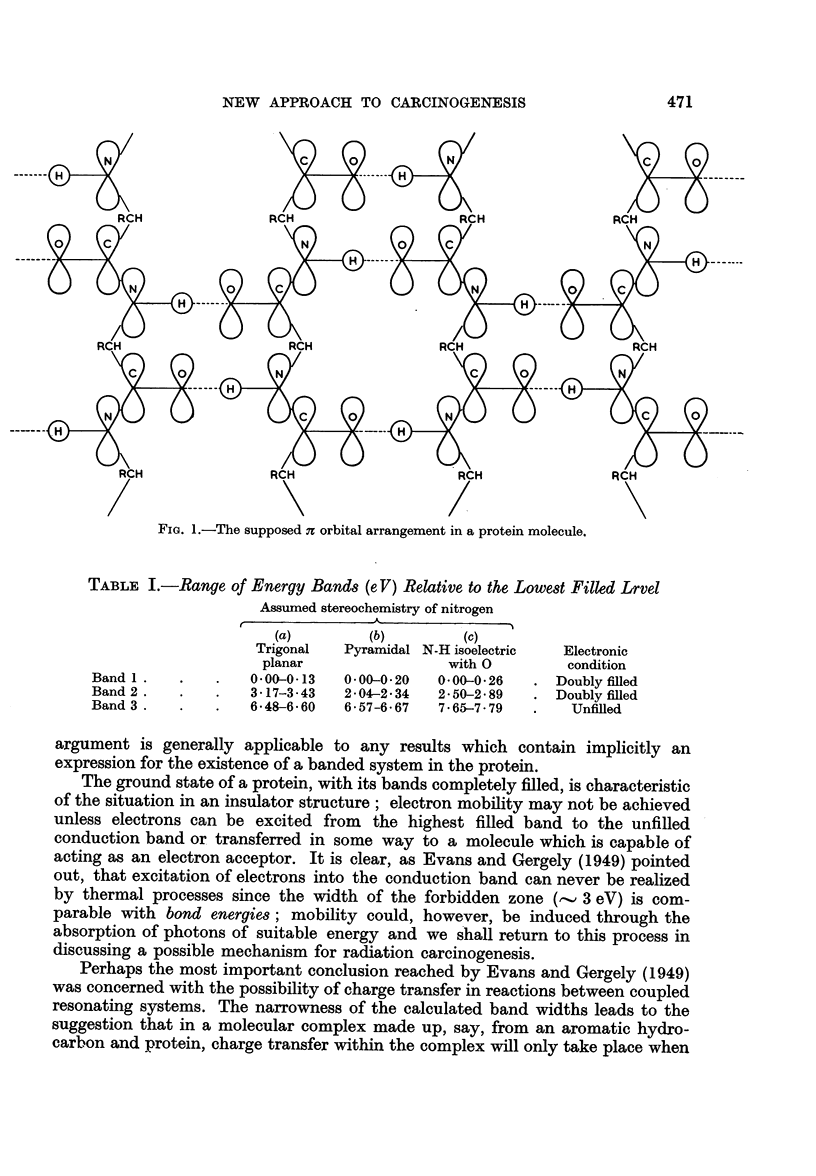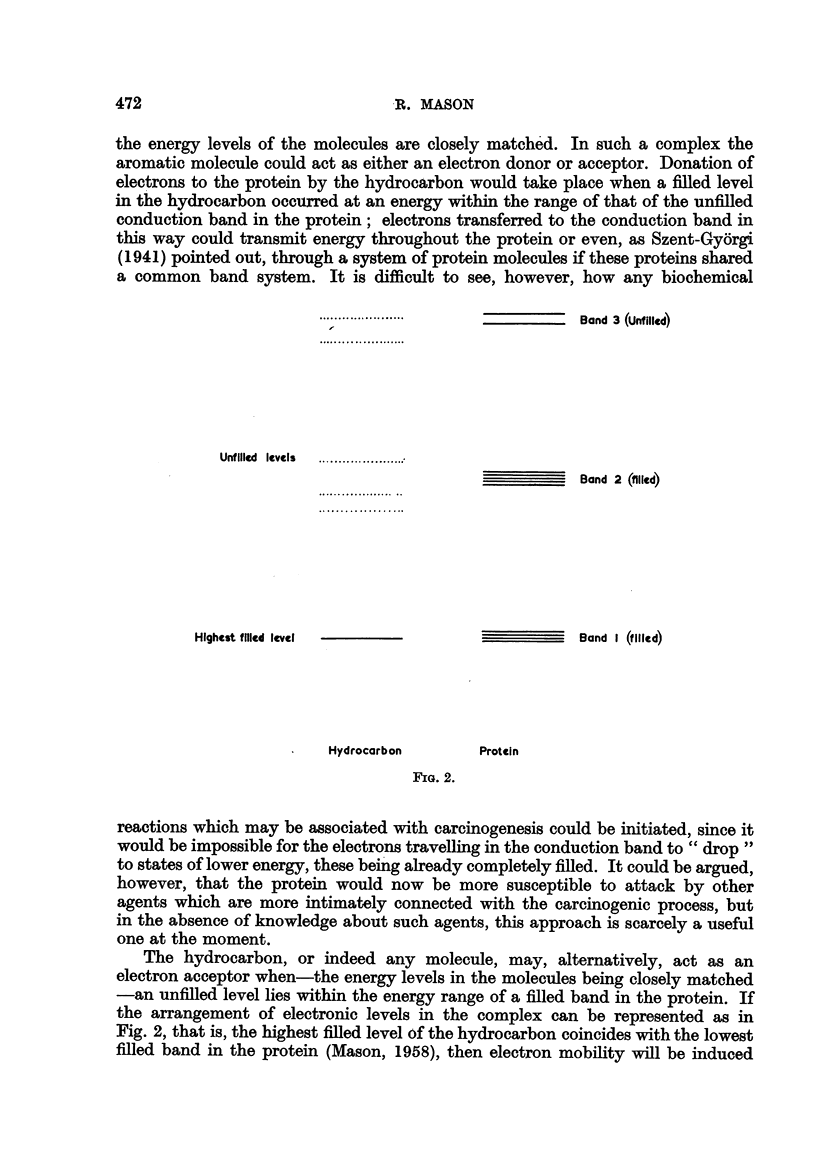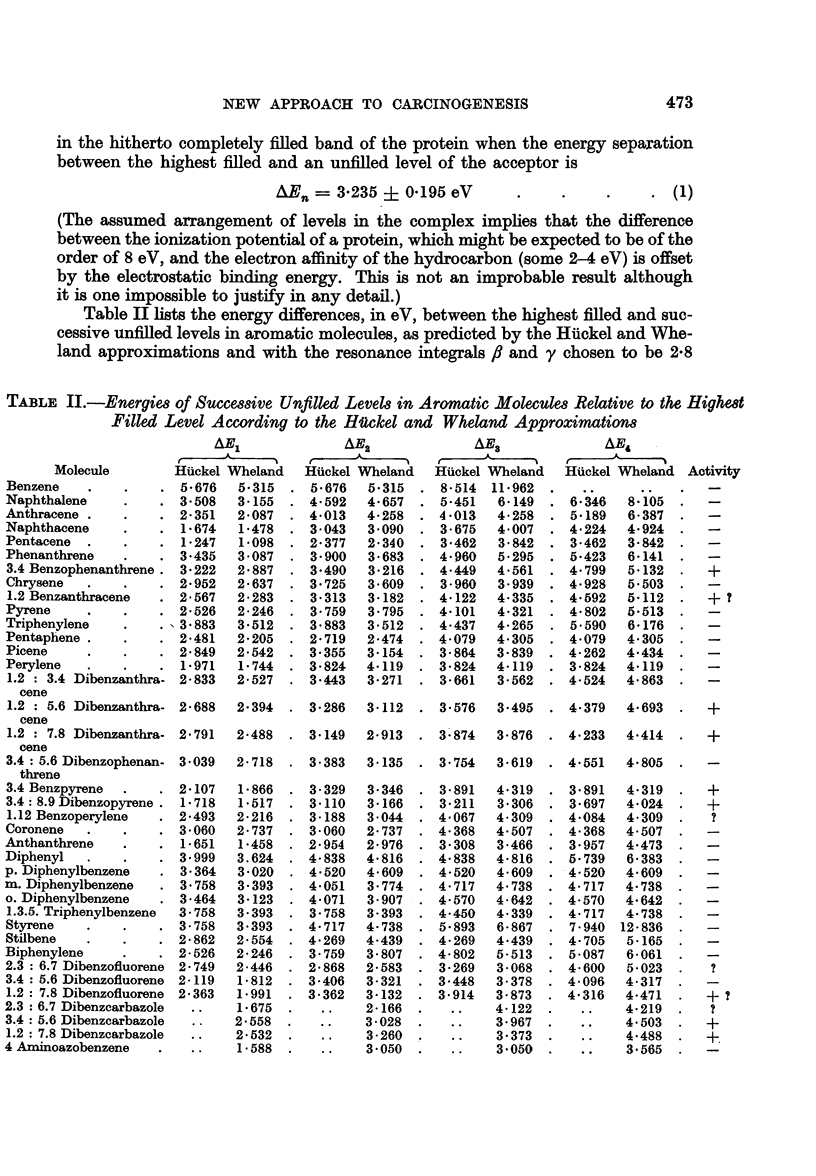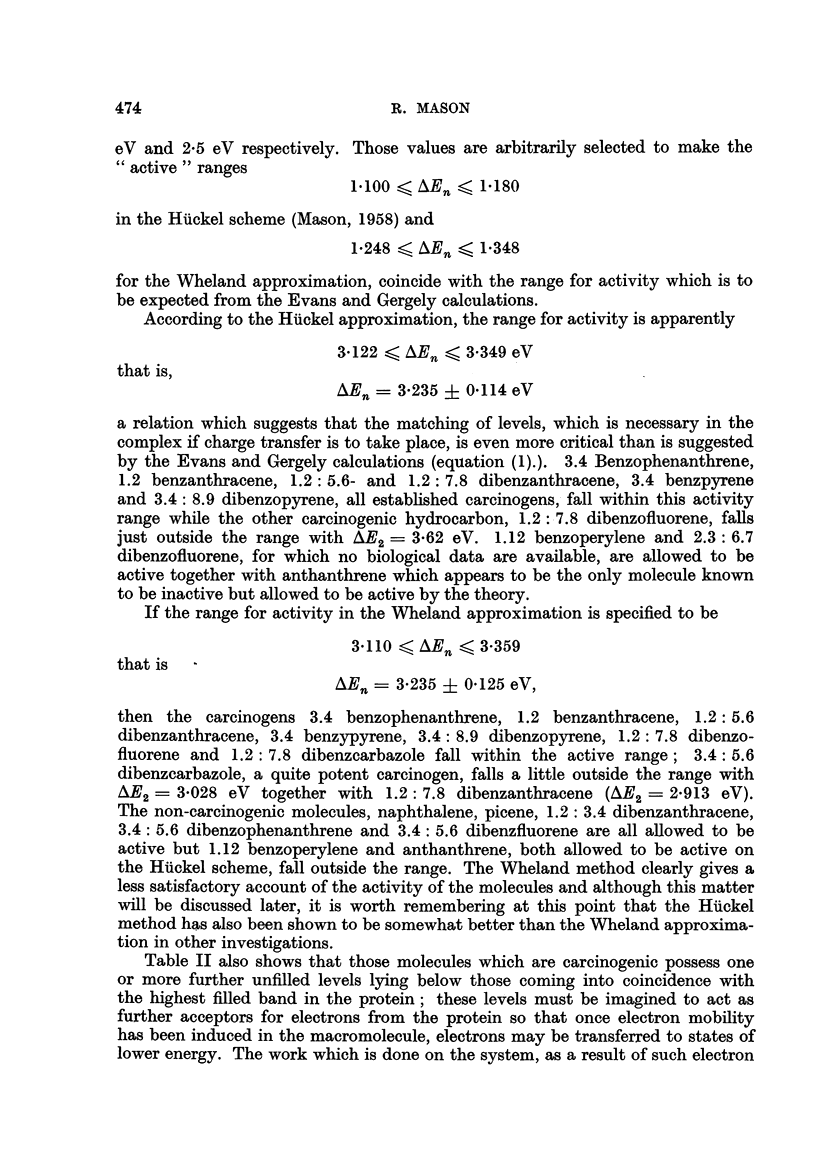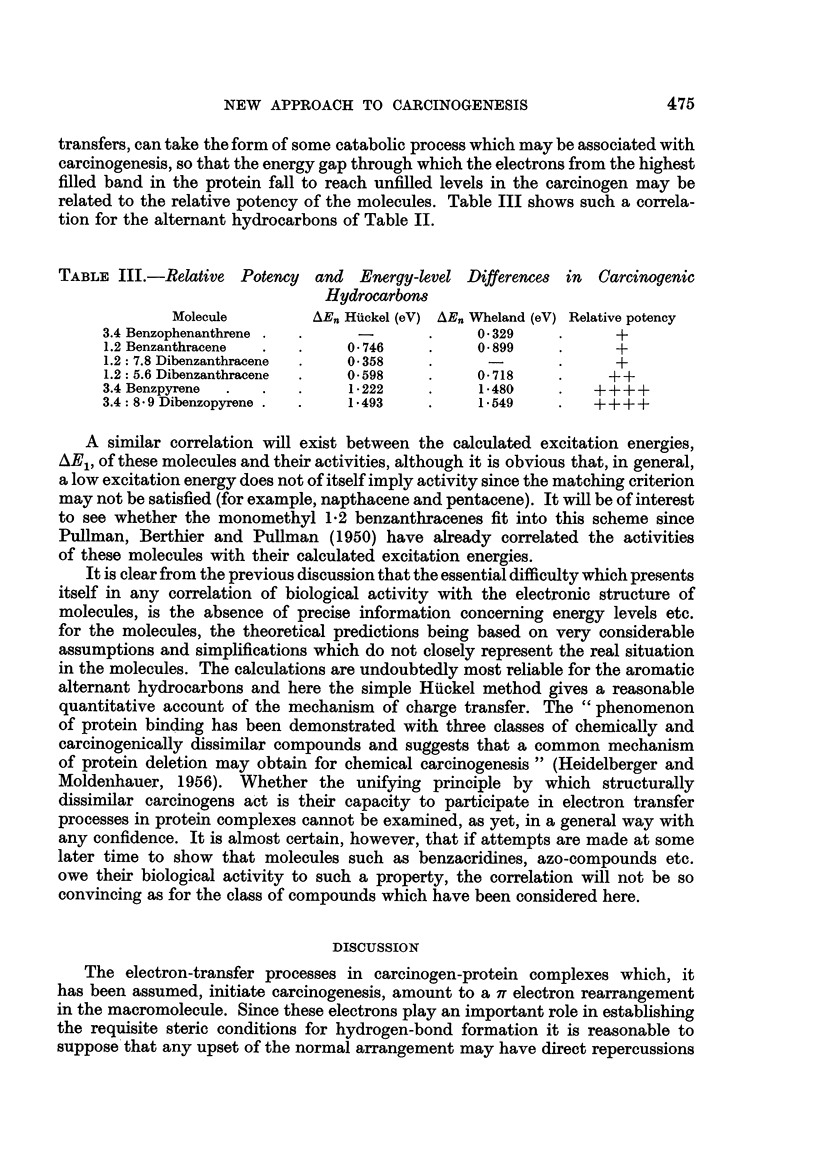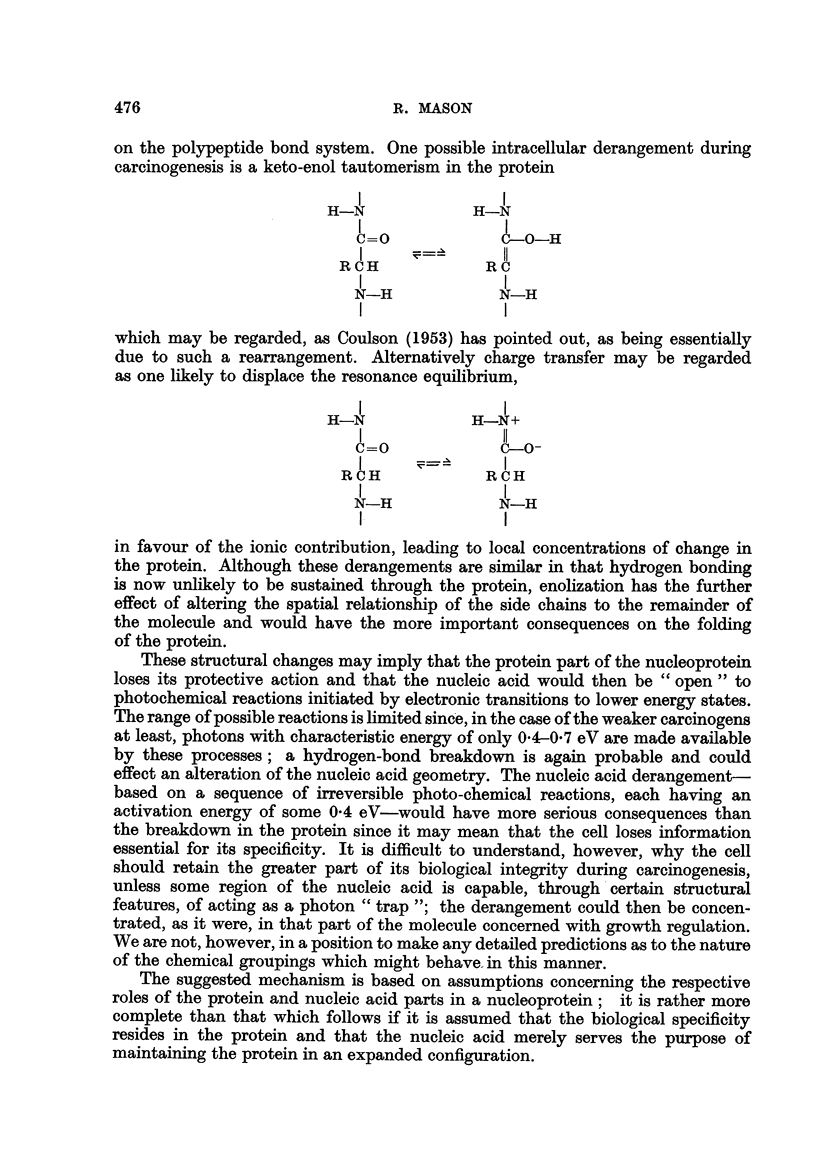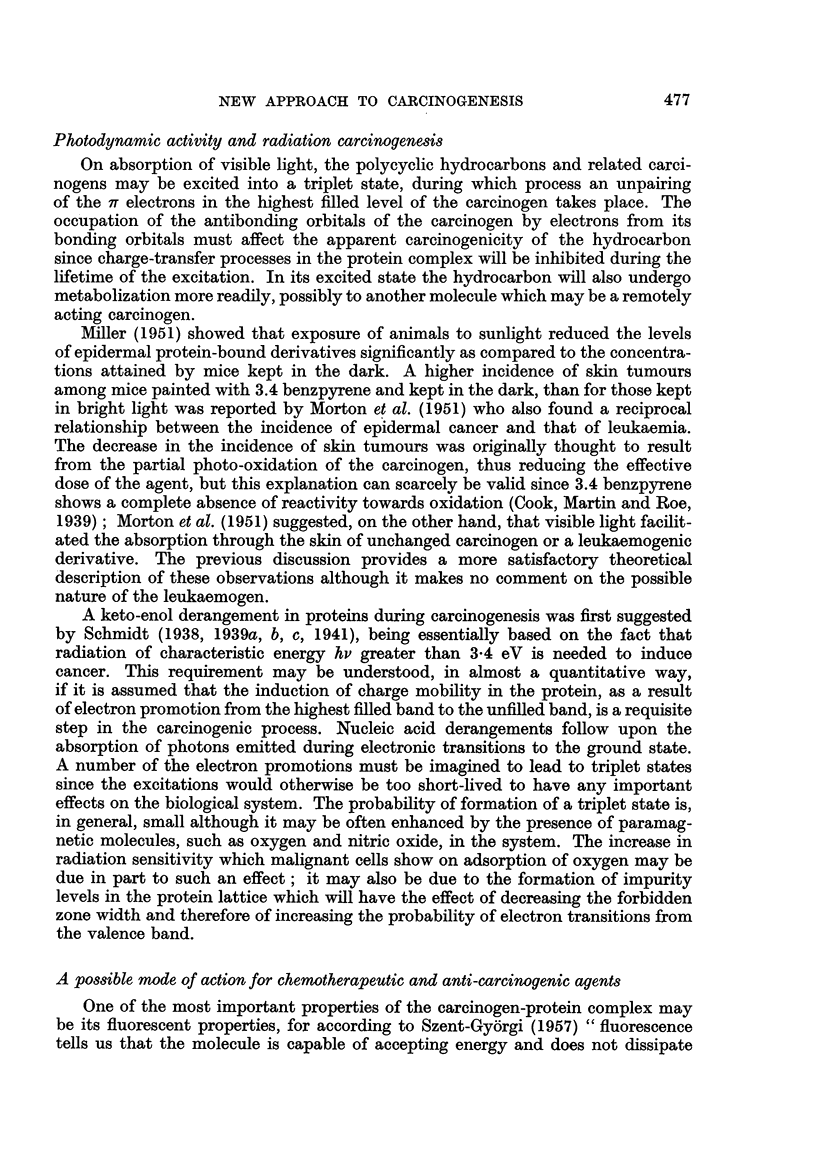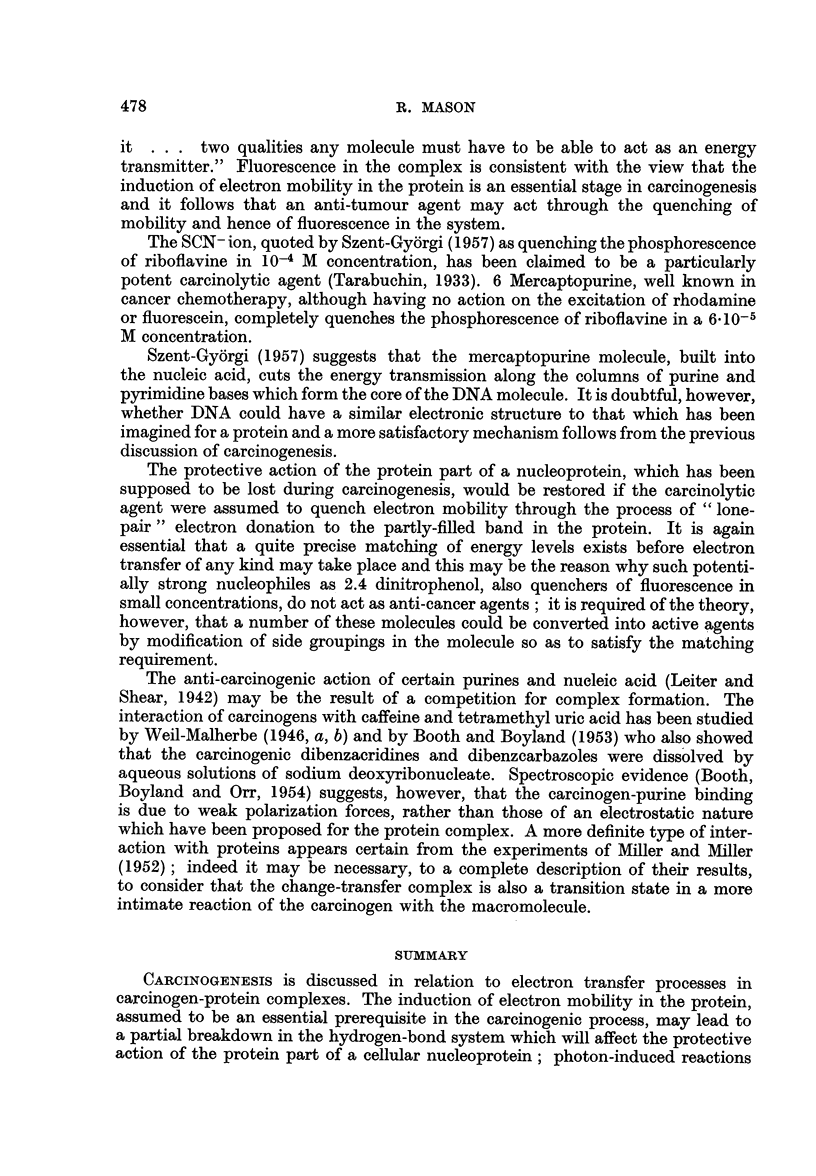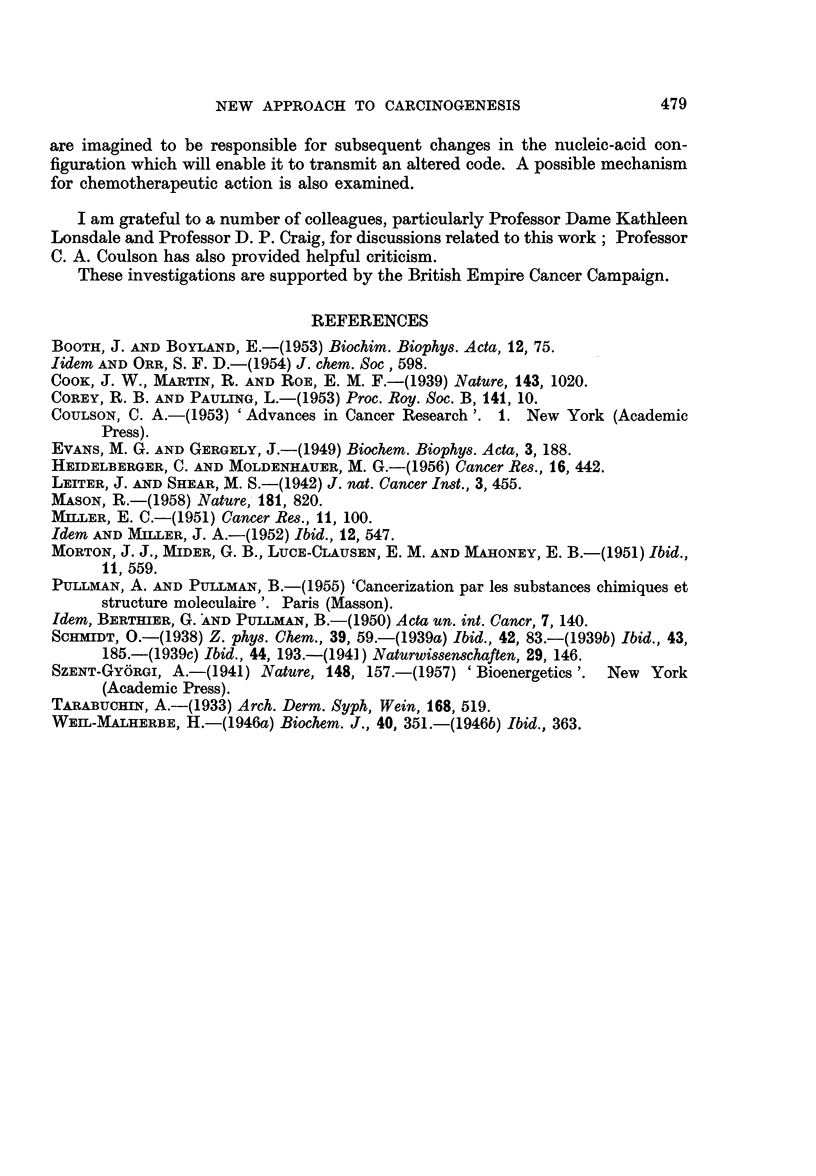# A New Approach to the Mechanism of Carcinogenesis

**DOI:** 10.1038/bjc.1958.55

**Published:** 1958-09

**Authors:** R. Mason


### A NEW APPROACH TO THE MECHANISM OF CARCINOGENESIS

R. MASON

*Department of Chemical Crystallography, University College, London W.C.1*

Received for publication July 25, 1958

The most complete account to date of the carcinogenicity of aromatic compounds is due to Pullman and Pullman (1955) ; it is essentially based on the assumption that the biological activity of a molecule is related to its chemical reactivity, so that the interaction of the carcinogen with the cellular receiver takes place at or through the reactive " *K* " region of the molecule. This assumption is justified in so far as a correlation exists between certain calculated electron characteristics of this region and the carcinogenic activity, but no convincing correlation has yet been shown between activity and those molecular properties which should reflect the corresponding changes in structure. It is also clear that any apparent correlation within the polycyclic hydrocarbons is meaningless when applied to another class of carcinogens, although a good deal of evidence has accumulated suggesting that a number of chemically dissimilar carcinogens act through some common principle of protein deletion. The " deletion " hypothesis (Miller and Miller, 1952), derived from the observed *in vivo* binding of aromatic hydrocarbons and related molecules to proteins (see for example Heidelberger and Moldenhauer, 1956), suggests that the interaction of the carcinogen with the protein leads to the deletion of proteins involved as enzymes essential for the control of growth ; the purpose of the present discussion is to examine a mechanism by which the carcinogen might achieve such an effect.

### *The Electronic Structure of Carcinogen-Protein Complexes*

To discuss the nature of the carcinogen-protein interaction, the possibility of charge transfer within the complex and other related effects, a knowledge of the electron distribution in the carcinogen and protein separately is necessary ; in both cases the method of " molecular orbitals " has been used and although detailed descriptions of the method have been given elsewhere (Coulson, 1953), its more relevant aspects, as we require them, will be briefly discussed.

For an aromatic hydrocarbon or related compound, we need to describe the molecule by specifying which molecular orbitals are allowed for the aromatic ($\pi$) electrons and what are their energies. To facilitate the solution of the secular equations which provide such information, several approximations are available ; the nature or reality of these approximations are not of much interest here but the results of two approaches—the Hückel and Wheland methods—will be used to examine the consistency of the theoretical predictions. The approximations are identical in that the ground (unexcited) state of a molecule possessing $n$ $\pi$ electrons is usually described by the $n/2$ bonding orbitals, each doubly filled, while the antibonding orbitals, into which electrons may be excited, are empty. In the particular case of the Hückel approximation, all the orbital energies are in the form,

$$E_j = \alpha + m_j\beta$$

where $\alpha$ is the Coulomb integral and $\beta$ the resonance integral. In the Wheland method, the energies are given by a similar expression

$$E_j = \alpha + k_j\gamma$$

where $\gamma$ is now a new resonance integral defined by

$$\gamma = \beta - S\alpha$$

$S$ is the overlap integral (usually given the value of 0·25) and $k_j$ is related to the previous $m_j$ by

$$k_j = \frac{m_j}{1 + Sm_j}$$

For the so-called alternant hydrocarbons, where there are no closed rings in the molecule with an odd number of carbon atoms, equal positive and negative values of $m_j$ occur together, with the positive values usually denoting the bonding orbitals. This symmetry in orbital coefficients does not extend to the Wheland method.

The energy difference between levels $i$ and $j$ will therefore be

$$\Delta E_{ij} = (m_i - m_j)\,\beta$$

in the Hückel scheme, and

$$\Delta E_{ij} = (k_i - k_j)\,\gamma$$

according to the Wheland approximation. The energy difference is therefore generally assumed to be independent of $\alpha$, the Coulomb integral, and is usually quoted in units of $\beta$ or $\gamma$, the resonance integrals.

For our electronic model of a protein we can return to Szent-Györgi's original suggestions (1941) which likened the situation in a protein molecule, on account of its size and regularity of architecture, to the situation in a crystal lattice where quantum-mechanical methods show that the energy levels appropriate to the individual atoms fuse into a banded system extending over the whole lattice. The usefulness of such a model to explain biochemical reactions apparently involving action at a distance is clear ; and such suggestive evidence as the role which the iron-containing proteins, cytochrome $a$, $b$ and $c$, play in the cell's oxidation processes, the liberation through photon absorption of carbon monoxide from a carbon monoxide-myoglobin complex etc., led Evans and Gergely (1949) to a theoretical examination of the possibility of bands of energy levels in proteins as a result of electronic interaction between the atomic $\pi$ orbitals in such an arrangement as is shown in Fig. 1. These authors confirmed that the electronic structure of a protein could be described by a banded system and their results are summarized in Table I.

The general conclusion regarding the existence of bands of energy levels is unaltered if the several possible stereo-chemical configurations for nitrogen are included separately in the calculations, although the most likely result is that based on the trigonal configuration (Corey and Pauling, 1953). The following discussion will relate to these values in particular, although once again the

FIG. 1.—The supposed $\pi$ orbital arrangement in a protein molecule.

TABLE I.—*Range of Energy Bands (eV) Relative to the Lowest Filled Lrvel*

| | Assumed stereochemistry of nitrogen | | | |
|---|---|---|---|---|
| | (*a*) Trigonal planar | (*b*) Pyramidal | (*c*) N-H isoelectric with O | Electronic condition |
| Band 1 . . . | 0·00–0·13 | 0·00–0·20 | 0·00–0·26 . | Doubly filled |
| Band 2 . . . | 3·17–3·43 | 2·04–2·34 | 2·50–2·89 . | Doubly filled |
| Band 3 . . . | 6·48–6·60 | 6·57–6·67 | 7·65–7·79 . | Unfilled |

argument is generally applicable to any results which contain implicitly an expression for the existence of a banded system in the protein.

The ground state of a protein, with its bands completely filled, is characteristic of the situation in an insulator structure ; electron mobility may not be achieved unless electrons can be excited from the highest filled band to the unfilled conduction band or transferred in some way to a molecule which is capable of acting as an electron acceptor. It is clear, as Evans and Gergely (1949) pointed out, that excitation of electrons into the conduction band can never be realized by thermal processes since the width of the forbidden zone ($\sim$ 3 eV) is comparable with *bond energies* ; mobility could, however, be induced through the absorption of photons of suitable energy and we shall return to this process in discussing a possible mechanism for radiation carcinogenesis.

Perhaps the most important conclusion reached by Evans and Gergely (1949) was concerned with the possibility of charge transfer in reactions between coupled resonating systems. The narrowness of the calculated band widths leads to the suggestion that in a molecular complex made up, say, from an aromatic hydrocarbon and protein, charge transfer within the complex will only take place when the energy levels of the molecules are closely matched. In such a complex the aromatic molecule could act as either an electron donor or acceptor. Donation of electrons to the protein by the hydrocarbon would take place when a filled level in the hydrocarbon occurred at an energy within the range of that of the unfilled conduction band in the protein ; electrons transferred to the conduction band in this way could transmit energy throughout the protein or even, as Szent-Györgi (1941) pointed out, through a system of protein molecules if these proteins shared a common band system. It is difficult to see, however, how any biochemical

FIG. 2.

reactions which may be associated with carcinogenesis could be initiated, since it would be impossible for the electrons travelling in the conduction band to " drop " to states of lower energy, these being already completely filled. It could be argued, however, that the protein would now be more susceptible to attack by other agents which are more intimately connected with the carcinogenic process, but in the absence of knowledge about such agents, this approach is scarcely a useful one at the moment.

The hydrocarbon, or indeed any molecule, may, alternatively, act as an electron acceptor when—the energy levels in the molecules being closely matched—an unfilled level lies within the energy range of a filled band in the protein. If the arrangement of electronic levels in the complex can be represented as in Fig. 2, that is, the highest filled level of the hydrocarbon coincides with the lowest filled band in the protein (Mason, 1958), then electron mobility will be induced in the hitherto completely filled band of the protein when the energy separation between the highest filled and an unfilled level of the acceptor is

$$\Delta E_n = 3{\cdot}235 \pm 0{\cdot}195 \text{ eV} \quad . \quad . \quad . \quad . \quad (1)$$

(The assumed arrangement of levels in the complex implies that the difference between the ionization potential of a protein, which might be expected to be of the order of 8 eV, and the electron affinity of the hydrocarbon (some 2–4 eV) is offset by the electrostatic binding energy. This is not an improbable result although it is one impossible to justify in any detail.)

Table II lists the energy differences, in eV, between the highest filled and successive unfilled levels in aromatic molecules, as predicted by the Hückel and Wheland approximations and with the resonance integrals $\beta$ and $\gamma$ chosen to be 2·8

TABLE II.—*Energies of Successive Unfilled Levels in Aromatic Molecules Relative to the Highest Filled Level According to the Hückel and Wheland Approximations*

| | $\Delta E_1$ | | $\Delta E_2$ | | $\Delta E_3$ | | $\Delta E_4$ | | |
|---|---|---|---|---|---|---|---|---|---|
| Molecule | Hückel | Wheland | Hückel | Wheland | Hückel | Wheland | Hückel | Wheland | Activity |
| Benzene | 5·676 | 5·315 | 5·676 | 5·315 | 8·514 | 11·962 | .. | .. | — |
| Naphthalene | 3·508 | 3·155 | 4·592 | 4·657 | 5·451 | 6·149 | 6·346 | 8·105 | — |
| Anthracene | 2·351 | 2·087 | 4·013 | 4·258 | 4·013 | 4·258 | 5·189 | 6·387 | — |
| Naphthacene | 1·674 | 1·478 | 3·043 | 3·090 | 3·675 | 4·007 | 4·224 | 4·924 | — |
| Pentacene | 1·247 | 1·098 | 2·377 | 2·340 | 3·462 | 3·842 | 3·462 | 3·842 | — |
| Phenanthrene | 3·435 | 3·087 | 3·900 | 3·683 | 4·960 | 5·295 | 5·423 | 6·141 | — |
| 3.4 Benzophenanthrene | 3·222 | 2·887 | 3·490 | 3·216 | 4·449 | 4·561 | 4·799 | 5·132 | + |
| Chrysene | 2·952 | 2·637 | 3·725 | 3·609 | 3·960 | 3·939 | 4·928 | 5·503 | — |
| 1.2 Benzanthracene | 2·567 | 2·283 | 3·313 | 3·182 | 4·122 | 4·335 | 4·592 | 5·112 | + ? |
| Pyrene | 2·526 | 2·246 | 3·759 | 3·795 | 4·101 | 4·321 | 4·802 | 5·513 | — |
| Triphenylene | 3·883 | 3·512 | 3·883 | 3·512 | 4·437 | 4·265 | 5·590 | 6·176 | — |
| Pentaphene | 2·481 | 2·205 | 2·719 | 2·474 | 4·079 | 4·305 | 4·079 | 4·305 | — |
| Picene | 2·849 | 2·542 | 3·355 | 3·154 | 3·864 | 3·839 | 4·262 | 4·434 | — |
| Perylene | 1·971 | 1·744 | 3·824 | 4·119 | 3·824 | 4·119 | 3·824 | 4·119 | — |
| 1.2 : 3.4 Dibenzanthracene | 2·833 | 2·527 | 3·443 | 3·271 | 3·661 | 3·562 | 4·524 | 4·863 | — |
| 1.2 : 5.6 Dibenzanthracene | 2·688 | 2·394 | 3·286 | 3·112 | 3·576 | 3·495 | 4·379 | 4·693 | + |
| 1.2 : 7.8 Dibenzanthracene | 2·791 | 2·488 | 3·149 | 2·913 | 3·874 | 3·876 | 4·233 | 4·414 | + |
| 3.4 : 5.6 Dibenzophenanthrene | 3·039 | 2·718 | 3·383 | 3·135 | 3·754 | 3·619 | 4·551 | 4·805 | — |
| 3.4 Benzpyrene | 2·107 | 1·866 | 3·329 | 3·346 | 3·891 | 4·319 | 3·891 | 4·319 | + |
| 3.4 : 8.9 Dibenzopyrene | 1·718 | 1·517 | 3·110 | 3·166 | 3·211 | 3·306 | 3·697 | 4·024 | + |
| 1.12 Benzoperylene | 2·493 | 2·216 | 3·188 | 3·044 | 4·067 | 4·309 | 4·084 | 4·309 | ? |
| Coronene | 3·060 | 2·737 | 3·060 | 2·737 | 4·368 | 4·507 | 4·368 | 4·507 | — |
| Anthanthrene | 1·651 | 1·458 | 2·954 | 2·976 | 3·308 | 3·466 | 3·957 | 4·473 | — |
| Diphenyl | 3·999 | 3.624 | 4·838 | 4·816 | 4·838 | 4·816 | 5·739 | 6·383 | — |
| p. Diphenylbenzene | 3·364 | 3·020 | 4·520 | 4·609 | 4·520 | 4·609 | 4·520 | 4·609 | — |
| m. Diphenylbenzene | 3·758 | 3·393 | 4·051 | 3·774 | 4·717 | 4·738 | 4·717 | 4·738 | — |
| o. Diphenylbenzene | 3·464 | 3·123 | 4·071 | 3·907 | 4·570 | 4·642 | 4·570 | 4·642 | — |
| 1.3.5. Triphenylbenzene | 3·758 | 3·393 | 3·758 | 3·393 | 4·450 | 4·339 | 4·717 | 4·738 | — |
| Styrene | 3·758 | 3·393 | 4·717 | 4·738 | 5·893 | 6·867 | 7·940 | 12·836 | — |
| Stilbene | 2·862 | 2·554 | 4·269 | 4·439 | 4·269 | 4·439 | 4·705 | 5·165 | — |
| Biphenylene | 2·526 | 2·246 | 3·759 | 3·807 | 4·802 | 5·513 | 5·087 | 6·061 | — |
| 2.3 : 6.7 Dibenzofluorene | 2·749 | 2·446 | 2·868 | 2·583 | 3·269 | 3·068 | 4·600 | 5·023 | ? |
| 3.4 : 5.6 Dibenzofluorene | 2·119 | 1·812 | 3·406 | 3·321 | 3·448 | 3·378 | 4·096 | 4·317 | — |
| 1.2 : 7.8 Dibenzofluorene | 2·363 | 1·991 | 3·362 | 3·132 | 3·914 | 3·873 | 4·316 | 4·471 | + ? |
| 2.3 : 6.7 Dibenzcarbazole | .. | 1·675 | .. | 2·166 | .. | 4·122 | .. | 4·219 | ? |
| 3.4 : 5.6 Dibenzcarbazole | .. | 2·558 | .. | 3·028 | .. | 3·967 | .. | 4·503 | + |
| 1.2 : 7.8 Dibenzcarbazole | .. | 2·532 | .. | 3·260 | .. | 3·373 | .. | 4·488 | + |
| 4 Aminoazobenzene | .. | 1·588 | .. | 3·050 | .. | 3·050 | .. | 3·565 | — |

eV and 2·5 eV respectively. Those values are arbitrarily selected to make the "active" ranges

$$1{\cdot}100 \leqslant \Delta E_n \leqslant 1{\cdot}180$$

in the Hückel scheme (Mason, 1958) and

$$1{\cdot}248 \leqslant \Delta E_n \leqslant 1{\cdot}348$$

for the Wheland approximation, coincide with the range for activity which is to be expected from the Evans and Gergely calculations.

According to the Hückel approximation, the range for activity is apparently

$$3{\cdot}122 \leqslant \Delta E_n \leqslant 3{\cdot}349 \text{ eV}$$

that is,

$$\Delta E_n = 3{\cdot}235 \pm 0{\cdot}114 \text{ eV}$$

a relation which suggests that the matching of levels, which is necessary in the complex if charge transfer is to take place, is even more critical than is suggested by the Evans and Gergely calculations (equation (1).). 3.4 Benzophenanthrene, 1.2 benzanthracene, 1.2 : 5.6- and 1.2 : 7.8 dibenzanthracene, 3.4 benzpyrene and 3.4 : 8.9 dibenzopyrene, all established carcinogens, fall within this activity range while the other carcinogenic hydrocarbon, 1.2 : 7.8 dibenzofluorene, falls just outside the range with $\Delta E_2 = 3{\cdot}62$ eV. 1.12 benzoperylene and 2.3 : 6.7 dibenzofluorene, for which no biological data are available, are allowed to be active together with anthanthrene which appears to be the only molecule known to be inactive but allowed to be active by the theory.

If the range for activity in the Wheland approximation is specified to be

$$3{\cdot}110 \leqslant \Delta E_n \leqslant 3{\cdot}359$$

that is

$$\Delta E_n = 3{\cdot}235 \pm 0{\cdot}125 \text{ eV},$$

then the carcinogens 3.4 benzophenanthrene, 1.2 benzanthracene, 1.2 : 5.6 dibenzanthracene, 3.4 benzypyrene, 3.4 : 8.9 dibenzopyrene, 1.2 : 7.8 dibenzofluorene and 1.2 : 7.8 dibenzcarbazole fall within the active range; 3.4 : 5.6 dibenzcarbazole, a quite potent carcinogen, falls a little outside the range with $\Delta E_2 = 3{\cdot}028$ eV together with 1.2 : 7.8 dibenzanthracene ($\Delta E_2 = 2{\cdot}913$ eV). The non-carcinogenic molecules, naphthalene, picene, 1.2 : 3.4 dibenzanthracene, 3.4 : 5.6 dibenzophenanthrene and 3.4 : 5.6 dibenzfluorene are all allowed to be active but 1.12 benzoperylene and anthanthrene, both allowed to be active on the Hückel scheme, fall outside the range. The Wheland method clearly gives a less satisfactory account of the activity of the molecules and although this matter will be discussed later, it is worth remembering at this point that the Hückel method has also been shown to be somewhat better than the Wheland approximation in other investigations.

Table II also shows that those molecules which are carcinogenic possess one or more further unfilled levels lying below those coming into coincidence with the highest filled band in the protein; these levels must be imagined to act as further acceptors for electrons from the protein so that once electron mobility has been induced in the macromolecule, electrons may be transferred to states of lower energy. The work which is done on the system, as a result of such electron transfers, can take the form of some catabolic process which may be associated with carcinogenesis, so that the energy gap through which the electrons from the highest filled band in the protein fall to reach unfilled levels in the carcinogen may be related to the relative potency of the molecules. Table III shows such a correlation for the alternant hydrocarbons of Table II.

TABLE III.—*Relative Potency and Energy-level Differences in Carcinogenic Hydrocarbons*

| Molecule | $\Delta E_n$ Hückel (eV) | $\Delta E_n$ Wheland (eV) | Relative potency |
|---|---|---|---|
| 3.4 Benzophenanthrene | — | 0·329 | + |
| 1.2 Benzanthracene | 0·746 | 0·899 | + |
| 1.2 : 7.8 Dibenzanthracene | 0·358 | — | + |
| 1.2 : 5.6 Dibenzanthracene | 0·598 | 0·718 | ++ |
| 3.4 Benzpyrene | 1·222 | 1·480 | ++++ |
| 3.4 : 8·9 Dibenzopyrene | 1·493 | 1·549 | ++++ |

A similar correlation will exist between the calculated excitation energies, $\Delta E_1$, of these molecules and their activities, although it is obvious that, in general, a low excitation energy does not of itself imply activity since the matching criterion may not be satisfied (for example, napthacene and pentacene). It will be of interest to see whether the monomethyl 1·2 benzanthracenes fit into this scheme since Pullman, Berthier and Pullman (1950) have already correlated the activities of these molecules with their calculated excitation energies.

It is clear from the previous discussion that the essential difficulty which presents itself in any correlation of biological activity with the electronic structure of molecules, is the absence of precise information concerning energy levels etc. for the molecules, the theoretical predictions being based on very considerable assumptions and simplifications which do not closely represent the real situation in the molecules. The calculations are undoubtedly most reliable for the aromatic alternant hydrocarbons and here the simple Hückel method gives a reasonable quantitative account of the mechanism of charge transfer. The " phenomenon of protein binding has been demonstrated with three classes of chemically and carcinogenically dissimilar compounds and suggests that a common mechanism of protein deletion may obtain for chemical carcinogenesis " (Heidelberger and Moldenhauer, 1956). Whether the unifying principle by which structurally dissimilar carcinogens act is their capacity to participate in electron transfer processes in protein complexes cannot be examined, as yet, in a general way with any confidence. It is almost certain, however, that if attempts are made at some later time to show that molecules such as benzacridines, azo-compounds etc. owe their biological activity to such a property, the correlation will not be so convincing as for the class of compounds which have been considered here.

### DISCUSSION

The electron-transfer processes in carcinogen-protein complexes which, it has been assumed, initiate carcinogenesis, amount to a $\pi$ electron rearrangement in the macromolecule. Since these electrons play an important role in establishing the requisite steric conditions for hydrogen-bond formation it is reasonable to suppose that any upset of the normal arrangement may have direct repercussions on the polypeptide bond system. One possible intracellular derangement during carcinogenesis is a keto-enol tautomerism in the protein which may be regarded, as Coulson (1953) has pointed out, as being essentially due to such a rearrangement. Alternatively charge transfer may be regarded as one likely to displace the resonance equilibrium, in favour of the ionic contribution, leading to local concentrations of change in the protein. Although these derangements are similar in that hydrogen bonding is now unlikely to be sustained through the protein, enolization has the further effect of altering the spatial relationship of the side chains to the remainder of the molecule and would have the more important consequences on the folding of the protein.

These structural changes may imply that the protein part of the nucleoprotein loses its protective action and that the nucleic acid would then be " open " to photochemical reactions initiated by electronic transitions to lower energy states. The range of possible reactions is limited since, in the case of the weaker carcinogens at least, photons with characteristic energy of only 0·4–0·7 eV are made available by these processes ; a hydrogen-bond breakdown is again probable and could effect an alteration of the nucleic acid geometry. The nucleic acid derangement—based on a sequence of irreversible photo-chemical reactions, each having an activation energy of some 0·4 eV—would have more serious consequences than the breakdown in the protein since it may mean that the cell loses information essential for its specificity. It is difficult to understand, however, why the cell should retain the greater part of its biological integrity during carcinogenesis, unless some region of the nucleic acid is capable, through certain structural features, of acting as a photon " trap "; the derangement could then be concentrated, as it were, in that part of the molecule concerned with growth regulation. We are not, however, in a position to make any detailed predictions as to the nature of the chemical groupings which might behave in this manner.

The suggested mechanism is based on assumptions concerning the respective roles of the protein and nucleic acid parts in a nucleoprotein ; it is rather more complete than that which follows if it is assumed that the biological specificity resides in the protein and that the nucleic acid merely serves the purpose of maintaining the protein in an expanded configuration.

*Photodynamic activity and radiation carcinogenesis*

On absorption of visible light, the polycyclic hydrocarbons and related carcinogens may be excited into a triplet state, during which process an unpairing of the $\pi$ electrons in the highest filled level of the carcinogen takes place. The occupation of the antibonding orbitals of the carcinogen by electrons from its bonding orbitals must affect the apparent carcinogenicity of the hydrocarbon since charge-transfer processes in the protein complex will be inhibited during the lifetime of the excitation. In its excited state the hydrocarbon will also undergo metabolization more readily, possibly to another molecule which may be a remotely acting carcinogen.

Miller (1951) showed that exposure of animals to sunlight reduced the levels of epidermal protein-bound derivatives significantly as compared to the concentrations attained by mice kept in the dark. A higher incidence of skin tumours among mice painted with 3.4 benzpyrene and kept in the dark, than for those kept in bright light was reported by Morton *et al.* (1951) who also found a reciprocal relationship between the incidence of epidermal cancer and that of leukaemia. The decrease in the incidence of skin tumours was originally thought to result from the partial photo-oxidation of the carcinogen, thus reducing the effective dose of the agent, but this explanation can scarcely be valid since 3.4 benzpyrene shows a complete absence of reactivity towards oxidation (Cook, Martin and Roe, 1939) ; Morton *et al.* (1951) suggested, on the other hand, that visible light facilitated the absorption through the skin of unchanged carcinogen or a leukaemogenic derivative. The previous discussion provides a more satisfactory theoretical description of these observations although it makes no comment on the possible nature of the leukaemogen.

A keto-enol derangement in proteins during carcinogenesis was first suggested by Schmidt (1938, 1939*a*, *b*, *c*, 1941), being essentially based on the fact that radiation of characteristic energy $h\nu$ greater than 3·4 eV is needed to induce cancer. This requirement may be understood, in almost a quantitative way, if it is assumed that the induction of charge mobility in the protein, as a result of electron promotion from the highest filled band to the unfilled band, is a requisite step in the carcinogenic process. Nucleic acid derangements follow upon the absorption of photons emitted during electronic transitions to the ground state. A number of the electron promotions must be imagined to lead to triplet states since the excitations would otherwise be too short-lived to have any important effects on the biological system. The probability of formation of a triplet state is, in general, small although it may be often enhanced by the presence of paramagnetic molecules, such as oxygen and nitric oxide, in the system. The increase in radiation sensitivity which malignant cells show on adsorption of oxygen may be due in part to such an effect ; it may also be due to the formation of impurity levels in the protein lattice which will have the effect of decreasing the forbidden zone width and therefore of increasing the probability of electron transitions from the valence band.

*A possible mode of action for chemotherapeutic and anti-carcinogenic agents*

One of the most important properties of the carcinogen-protein complex may be its fluorescent properties, for according to Szent-Györgi (1957) " fluorescence tells us that the molecule is capable of accepting energy and does not dissipate it . . . two qualities any molecule must have to be able to act as an energy transmitter." Fluorescence in the complex is consistent with the view that the induction of electron mobility in the protein is an essential stage in carcinogenesis and it follows that an anti-tumour agent may act through the quenching of mobility and hence of fluorescence in the system.

The $SCN^-$ ion, quoted by Szent-Györgi (1957) as quenching the phosphorescence of riboflavine in $10^{-4}$ M concentration, has been claimed to be a particularly potent carcinolytic agent (Tarabuchin, 1933). 6 Mercaptopurine, well known in cancer chemotherapy, although having no action on the excitation of rhodamine or fluorescein, completely quenches the phosphorescence of riboflavine in a $6 \cdot 10^{-5}$ M concentration.

Szent-Györgi (1957) suggests that the mercaptopurine molecule, built into the nucleic acid, cuts the energy transmission along the columns of purine and pyrimidine bases which form the core of the DNA molecule. It is doubtful, however, whether DNA could have a similar electronic structure to that which has been imagined for a protein and a more satisfactory mechanism follows from the previous discussion of carcinogenesis.

The protective action of the protein part of a nucleoprotein, which has been supposed to be lost during carcinogenesis, would be restored if the carcinolytic agent were assumed to quench electron mobility through the process of " lone-pair " electron donation to the partly-filled band in the protein. It is again essential that a quite precise matching of energy levels exists before electron transfer of any kind may take place and this may be the reason why such potentially strong nucleophiles as 2.4 dinitrophenol, also quenchers of fluorescence in small concentrations, do not act as anti-cancer agents ; it is required of the theory, however, that a number of these molecules could be converted into active agents by modification of side groupings in the molecule so as to satisfy the matching requirement.

The anti-carcinogenic action of certain purines and nucleic acid (Leiter and Shear, 1942) may be the result of a competition for complex formation. The interaction of carcinogens with caffeine and tetramethyl uric acid has been studied by Weil-Malherbe (1946, *a*, *b*) and by Booth and Boyland (1953) who also showed that the carcinogenic dibenzacridines and dibenzcarbazoles were dissolved by aqueous solutions of sodium deoxyribonucleate. Spectroscopic evidence (Booth, Boyland and Orr, 1954) suggests, however, that the carcinogen-purine binding is due to weak polarization forces, rather than those of an electrostatic nature which have been proposed for the protein complex. A more definite type of interaction with proteins appears certain from the experiments of Miller and Miller (1952) ; indeed it may be necessary, to a complete description of their results, to consider that the change-transfer complex is also a transition state in a more intimate reaction of the carcinogen with the macromolecule.

### SUMMARY

Carcinogenesis is discussed in relation to electron transfer processes in carcinogen-protein complexes. The induction of electron mobility in the protein, assumed to be an essential prerequisite in the carcinogenic process, may lead to a partial breakdown in the hydrogen-bond system which will affect the protective action of the protein part of a cellular nucleoprotein ; photon-induced reactions are imagined to be responsible for subsequent changes in the nucleic-acid configuration which will enable it to transmit an altered code. A possible mechanism for chemotherapeutic action is also examined.

I am grateful to a number of colleagues, particularly Professor Dame Kathleen Lonsdale and Professor D. P. Craig, for discussions related to this work ; Professor C. A. Coulson has also provided helpful criticism.

These investigations are supported by the British Empire Cancer Campaign.

### REFERENCES

BOOTH, J. AND BOYLAND, E.—(1953) *Biochim. Biophys. Acta*, **12**, 75.

*Iidem* AND ORR, S. F. D.—(1954) *J. chem. Soc* , 598.

COOK, J. W., MARTIN, R. AND ROE, E. M. F.—(1939) *Nature*, **143**, 1020.

COREY, R. B. AND PAULING, L.—(1953) *Proc. Roy. Soc.* B, **141**, 10.

COULSON, C. A.—(1953) ' Advances in Cancer Research '. **1**. New York (Academic Press).

EVANS, M. G. AND GERGELY, J.—(1949) *Biochem. Biophys. Acta*, **3**, 188.

HEIDELBERGER, C. AND MOLDENHAUER, M. G.—(1956) *Cancer Res.*, **16**, 442.

LEITER, J. AND SHEAR, M. S.—(1942) *J. nat. Cancer Inst.*, **3**, 455.

MASON, R.—(1958) *Nature*, **181**, 820.

MILLER, E. C.—(1951) *Cancer Res.*, **11**, 100.

*Idem* AND MILLER, J. A.—(1952) *Ibid.*, **12**, 547.

MORTON, J. J., MIDER, G. B., LUCE-CLAUSEN, E. M. AND MAHONEY, E. B.—(1951) *Ibid.*, **11**, 559.

PULLMAN, A. AND PULLMAN, B.—(1955) 'Cancerization par les substances chimiques et structure moleculaire '. Paris (Masson).

*Idem*, BERTHIER, G. AND PULLMAN, B.—(1950) *Acta un. int. Cancr*, **7**, 140.

SCHMIDT, O.—(1938) *Z. phys. Chem.*, **39**, 59.—(1939*a*) *Ibid.*, **42**, 83.—(1939*b*) *Ibid.*, **43**, 185.—(1939*c*) *Ibid.*, **44**, 193.—(1941) *Naturwissenschaften*, **29**, 146.

SZENT-GYÖRGI, A.—(1941) *Nature*, **148**, 157.—(1957) ' Bioenergetics '. New York (Academic Press).

TARABUCHIN, A.—(1933) *Arch. Derm. Syph, Wein*, **168**, 519.

WEIL-MALHERBE, H.—(1946*a*) *Biochem. J.*, **40**, 351.—(1946*b*) *Ibid.*, 363.